# Diagnosis and management of eosinophilic esophagitis and esophageal food impaction in adults: A position paper issued by the Austrian Society of Gastroenterology and Hepatology (ÖGGH)

**DOI:** 10.1007/s00508-024-02401-w

**Published:** 2024-09-04

**Authors:** Hansjörg Schlager, Franziska Baumann-Durchschein, Karin Steidl, Michael Häfner, Patrick Dinkhauser, Michael Weitersberger, Josef Holzinger, Markus Mader, Hans Peter Gröchenig, Christian Madl, Philipp Schreiner

**Affiliations:** 1 Division of Gastroenterology and Hepatology, Department of Internal Medicine, Medical University of Graz, University Hospital Graz, Auenbruggerplatz 15, 8036 Graz, Austria; 2 Department of Internal Medicine, Barmherzige Brüder St. Veit/Glan, St. Veit, Austria; 3 2nd Medical Department, Barmherzige Schwestern Krankenhaus, Vienna, Austria; 4 Department of Internal Medicine I, Division of Gastroenterology and Hepatology, Endocrinology and Rheumatology, Klinikum Wels-Grieskirchen, Wels, Austria; 5 Department of Gastroenterology and Hepatology, Ordensklinikum Linz Barmherzige Schwestern, Linz, Austria; 6 Department of Surgery, Paracelsus Medical University/Salzburger Landeskliniken (SALK), Salzburg, Austria; 7 Department of Internal Medicine II, Universitätsklinikum St. Pölten—Karl Landsteiner Privatuniversität, St. Pölten, Austria; 8 Division of Gastroenterology and Hepatology, Krankenanstalt Rudolfstiftung, Krankenanstaltenverbund Wien (KAV), Vienna, Austria; 9 Division of Gastroenterology and Hepatology, Department of Internal Medicine III, Medical University of Vienna, Vienna, Austria

**Keywords:** EoE, EFI, TH2-Inflammation, Eosinophile Ösophagitis, Ösophageale Bolusimpaktierung

## Abstract

This position paper deals with an expert consensus on diagnosis and management of eosinophilic esophagitis and esophageal food impaction issued by the Austrian Eosinophilic Esophagitis Network, a working group under the patronage of the Austrian Society of Gastroenterology and Hepatology (ÖGGH). In need of a standardized approach on the management of EoE, recommendations were made based on international guidelines and landmark studies.

## Summary of recommendations

### Diagnosis

#### Recommendation 1.1

In adult patients with esophageal symptoms, especially dysphagia, PPI-refractory symptoms and non-cardiac chest pain, an esophagogastroduodenoscopy (EGD) should be performed.

#### Recommendation 1.2

Atopic diathesis, IgE-mediated food allergy and other signs of TH2 inflammation (atopic dermatitis, asthma, chronic rhinosinusitis and pollinosis) should be systematically documented.

#### Recommendation 1.3

An oral allergen immunotherapy may induce or exacerbate EoE and should be actively asked for.

#### Recommendation 1.4

Esophageal biopsies must be taken even in cases of a macroscopically normal esophagus. If endoscopic features are present, directed biopsies of this lesions should be performed due to higher eosinophilic counts in these areas. At least six biopsies should be taken, including three biopsies from the distal and three biopsies from middle/proximal esophagus. The distal biopsies should not include the transition zone.

#### Recommendation 1.5

As treatment with proton pump inhibitors may mask EoE, patients should be actively asked about the PPI intake in the last weeks prior to diagnostic endoscopy.

#### Recommendation 1.6

At index endoscopy, additional biopsies (at least eight biopsies of the stomach and four biopsies of the duodenum) should be taken to rule out eosinophilic gastritis and/or eosinophilic duodenitis, especially in patients with diarrhea, abdominal pain or bloating.

#### Recommendation 1.7

Diagnostic criteria for EoE are symptoms of esophageal dysfunction and esophageal eosinophilia (mucosal and/or submucosal). The threshold for the histological diagnosis of EoE is 15 eos/hpf (standard size of ∼0.3 mm^2^) in any biopsy specimen.

#### Recommendation 1.8

In cases of clinical suspicion of EoE but not fulfilling the histological criteria of at least 15 eos/hpf, the biopsy samples should be re-evaluated by an expert in GI pathology. A sampling error should be suspected as well as a potentially underlying EoE variant.

#### Recommendation 1.9

Endoscopic features of EoE are edema, rings, exudates, furrows and strictures. These signs should be evaluated with the EREFS score. The most affected area should be documented in the endoscopy report.

#### Recommendation 1.10

Allergy testing with a skin prick test (SPT), atopy patch test (APT) or antigen-specific IgE antibodies is not recommended to identify the culprit food allergen.

#### Recommendation 1.11

Blood or urinary tests in eosinophilic esophagitis are not generally recommended due to low sensitivity or specificity for diagnosis of eosinophilic esophagitis.

#### Recommendation 1.12

EoE and GERD are two distinct disease entities that can co-exist in one individual patient and can impact each other.

### Therapy

#### Recommendation 2.1

In all patients with the diagnosis of EoE, an induction therapy should be started.

#### Recommendation 2.2

Therapeutic options for treating EoE are swallowed topical corticosteroids (STC), high dose proton pump inhibitors, dupilumab or elimination diet.

#### Recommendation 2.2.1

Swallowed topical corticosteroids (STC) are effective in achieving clinical histological remission and may be considered as first line induction therapy for 6–12 weeks. As maintenance therapy, STCs can be safely used as long-term treatment.

#### Recommendation 2.2.2

In patients with simultaneous GERD or other indications for acid suppression, PPI should be considered as induction and maintenance therapy.

#### Recommendation 2.2.3

Elimination diet can be considered as induction and maintenance treatment. If this treatment option is considered, a consultation with a dietician is mandatory. Due to less restriction and improvement of quality of life a 1FED (animal milk) or a step-up approach with 2FED (animal milk and wheat/gluten) is preferred.

#### Recommendation 2.2.4

Dupilumab can be considered as induction and maintenance treatment in EoE. Mostly used in patients refractory or intolerant to STC, it may be used as first line therapy in patients with other clinically relevant type 2 inflammatory diseases or contraindications for corticosteroids.

#### Recommendation 2.3

After 12 weeks of induction therapy, an endoscopic follow-up with biopsies is necessary to demonstrate histologic response.

#### Recommendation 2.4

In order to avoid long-term complications such as fibrosis, to improve quality of life and to reduce the risk of esophageal food impaction, maintenance therapy is mandatory.

#### Recommendation 2.5

In the majority of patients, combination therapies cannot be recommended. This option can be considered in selected cases with partial response to the first therapy.

#### Recommendation 2.6

After every major change of treatment, a clinical and histological re-evaluation should be performed to ensure maintenance of remission.

#### Recommendation 2.7

Due to high risk of recurrence and esophageal food impaction, a trial of withdrawal of treatment is not recommended.

#### Recommendation 2.8

Patients with STC-refractory disease should be evaluated and managed in institutions experienced with management of EoE.

### Dilation

#### Recommendation 3.1

In patients with clinically relevant fibrostenosis and with the necessity of dilation, a combination with anti-inflammatory treatment is required.

#### Recommendation 3.2

Regardless of the used technique, esophageal dilation is safe and effective in patients with EoE.

#### Recommendation 3.3

In patients with persistent dysphagia despite histological remission and lack of a visible stricture an empirical dilation may be performed.

#### Recommendation 3.4

If dilation is necessary, a luminal diameter of ≥ 16 mm should be aimed for to reduce long-term complications.

### Esophageal food impaction

#### Recommendation 4.1

Patients with EFI should not induce vomiting and should preferably attend an endoscopy unit/emergency department within 2 h.

#### Recommendation 4.2

Patients with EFI should preferably undergo esophagogastroduodenoscopy within 6 h. Before EGD, radiological evaluation or medications to resolve EFI is not advisable.

#### Recommendation 4.3

There is lacking evidence whether general anesthesia with endotracheal intubation or conscious sedation should be used in cases of EFI.

#### Recommendation 4.4

Push or pull technique with or without endoscopic devices may be used depending on physicians’ expertise.

#### Recommendation 4.5

In patients presenting with EFI, sufficient biopsies of the esophagus should be taken during the index endoscopy to diagnose or exclude eosinophilic esophagitis.

#### Recommendation 4.6

In patients with spontaneous resolution of EFI or in patients where sufficient esophageal biopsies have not been performed during index endoscopy, an endoscopy without PPI or STC should be repeated within 2–3 weeks.

#### Recommendation 4.7

Any patient presenting with EFI to the emergency department should receive an appointment within the next 4 weeks in the gastroenterology outpatient clinic.

### Follow-up

#### Recommendation 5.1

Patients with EoE in clinicopathological remission should have clinical and endoscopic follow-up every 1–2 years.

## Introduction

Eosinophilic esophagitis (EoE) is a chronic immune-mediated disease with an increasing incidence and a prevalence of 75 cases per 100,000 inhabitant-years [1]. Despite an increasing understanding of EoE, the delay from symptoms to diagnosis is still around 4 years [2] and care providers often neglect the recommendations of guidelines [3]. Until a few years ago, there was no approved drug for EoE, which has now changed with budesonide orodispersible tablets [4] and just recently, with dupilumab [5], the first biologic that has been approved for EoE. Proton pump inhibitors (PPIs) continue to be widely used as off-label therapy and diet remains the only treatment option that addresses the disease at its roots. In order to avoid long-term complications and to improve quality of life, a long-term management including maintenance therapy is necessary.

Esophageal food impaction (EFI) is an increasing entity with EoE being the most common cause nowadays [6]; however, the existing guidelines from 2016 (ESGE: removal of foreign bodies in the upper gastrointestinal tract in adults) [7] and 2011 (ASGE: management of ingested foreign bodies and food impactions) hardly discuss the management of EFI [8]. Many important questions remain unanswered in these guidelines including pre-endoscopy management, the optimal management in the ER or postprocedural follow-up.

Due to the current lack of any guidelines, the management of EFI nowadays varies widely between countries and between centers. Additionally, a recently performed survey among more than 300 gastroenterologists from Switzerland, Europe and USA demonstrated that “best practice in management of EFI” is needed but difficult to obtain [9]. This guideline could harmonize the treatment of EFI and prevent the high rates of patients lost to follow-up after EFI.

## Diagnosis

### Recommendation 1.1

In adult patients with esophageal symptoms, especially dysphagia, PPI-refractory symptoms and non-cardiac chest pain, an esophagogastroduodenoscopy (EGD) should be performed.

Although the cardinal symptom of adult patients with EoE is solid food dysphagia, other unspecific symptoms such as PPI-refractory burning sensation, non-cardiac chest pain or painful sensation rapidly after swallowing (FIRE: food-induced immediate response of the esophagus) [10] may occur in patients with EoE. In contrast to adults, children with EoE present more often with abdominal pain, diarrhea and growth retardation [11].

### Recommendation 1.2

Atopic diathesis, IgE-mediated food allergy and other signs of TH2 inflammation (atopic dermatitis, asthma, chronic rhinosinusitis and pollinosis) should be documented systematically.

EoE is a chronic immune-mediated disease with a TH2 type inflammatory response [12]. It is considered as a late manifestation of the allergic march [13] and the majority of patients will have other concomitant atopic conditions [14]. Although EoE is not an IgE-mediated disease, around half of the patients with EoE have an IgE-related food allergy (FA) [15] and vice versa, 5% of patients with an IgE-related FA have EoE [16]. Besides immunological and environmental factors, there exists a strong genetic association with asthma and atopic dermatitis [17].

Nowadays, dupilumab, an antibody directed against the interleukin‑4 receptor subunit α (IL-4Rα), targeting other type 2 inflammatory diseases such as asthma and atopic dermatitis, is approved for the treatment of EoE. Therefore, it is crucial to be aware of these comorbidities in the same patient to guide optimal treatment selection.

### Recommendation 1.3

An oral allergen immunotherapy may induce or exacerbate EoE and should be actively questioned.

Oral allergen immunotherapy (OIT) is an emerging therapeutic option in patients with IgE-mediated FA and is approved by the European Medicines Agency (EMA). An OIT may induce and exacerbate pre-existing esophageal eosinophilia [18] and result in a new diagnosis of EoE (in patients having symptoms of esophageal dysfunction). Although it could be shown that some asymptomatic patients with IgE-mediated FA have esophageal eosinophilia [19], it is unusual to perform EGD in asymptomatic patients before OIT. Therefore, it is not clear whether this OIT-induced EoE was pre-existing at a very mild clinical level or whether OIT induced EoE de novo.

Although most patients will have complete resolution of symptoms and esophageal eosinophilia after discontinuation of OIT [20], EoE may persist in a minority of patients [21]. Therefore, OIT should be actively asked for because this patient group may be candidates for a therapy outlet attempt after discontinuing the immunotherapy.

### Recommendation 1.4

Esophageal biopsies must be taken even in case of a macroscopically normal esophagus. If endoscopic features are present, directed biopsies of this lesions should be performed due to higher eosinophilic counts in these areas. At least six biopsies should be taken, including three biopsies from the distal and three biopsies from middle/proximal esophagus. The distal biopsies should not include the transition zone.

As EoE is a patchy disease, a higher number of biopsy samples increases the diagnostic yield. The sensitivity of a correct diagnostic of EoE increases from 55% with 1 biopsy to 100% when obtaining 5 or more biopsy specimens [22].

Although data suggest obtaining biopsies from multiple levels, it is unclear whether biopsies should be placed in different containers. It is more important to obtain biopsies from typical endoscopic features of active inflammation such as edema, furrows and exudates due to higher eosinophilic infiltrates in these areas [23]. In approximately 7–10% of patients with EoE the esophagus appears macroscopically normal [24]. Therefore, biopsies should be taken regardless of the endoscopic appearance in patients with symptoms of esophageal dysfunction. Distal biopsies should be obtained 3 cm above the gastroesophageal junction.

### Recommendation 1.5

As treatment with proton pump inhibitors may mask EoE, patients should be ctively asked about the PPI intake in the last weeks prior to diagnostic endoscopy.

Proton pump inhibitors (PPI) exert an anti-inflammatory effect in blocking the secretion of eotaxin‑3 and subsequently eliminate eosinophils from the esophageal epithelium. As there is neither a clinical, endoscopic nor histological difference between patients with a PPI-responsive eosinophilic esophagitis (PPI-REE) and patients without a PPI responsiveness, the term PPI-REE should not be used anymore. Nowadays, PPI are considered as one of many options to treat EoE [25]. Hence, a treatment with PPI may obscure endoscopic and histological signs of EoE [26]. Therapy with PPI should therefore be stopped prior to a diagnostic endoscopy. Although no data exist regarding the duration of action after stopping PPI, we advocate to stop PPI at least 3 weeks before the diagnostic endoscopy.

### Recommendation 1.6

At index endoscopy additional biopsies (at least eight biopsies of the stomach and four biopsies of the duodenum) should be taken to rule out eosinophilic gastritis and/or eosinophilic duodenitis, especially in patients with diarrhea, abdominal pain or bloating.

Without a secondary cause of eosinophilia, eosinophilic gastritis (EoG), enteritis (EoN) and colitis (EoC) can be summarized under the umbrella term of non-EoE gastrointestinal diseases (non-EGID) [27]. These non-EoE EGID may present with different unspecific symptoms such as nausea, diarrhea, abdominal pain and bloating [28]. Additionally, at least fecal calprotectin should be tested and colonoscopy should be considered in these patients. Due to the lack of data, it is debatable to name it EoG (or other non-EoE EGID) and EoE or EoG with esophageal involvement [27]. In most of these cases other GI symptoms are dominant and dysphagia is only a minor complaint. In order to treat the correct part of the EGID, it is important to rule out an additional non-EoE EGID in patients with unspecific GI symptoms. Similar to EoE, EoG and EoN are patchy diseases that need multiple biopsies to increase the diagnostic yield. Recent data suggest that at least eight biopsies of the stomach and four in the duodenum should be taken [29].

### Recommendation 1.7

Diagnostic criteria for EoE are symptoms of esophageal dysfunction and esophageal eosinophilia (mucosal and/or submucosal). The threshold for the histologic diagnosis of EoE is 15 eos/hpf (standard size of ∼0.3 mm^2^) in any biopsy specimen.

The diagnosis of EoE contains of a clinical part with symptoms of esophageal dysfunction and a histological part with more or equal than 15 intraepithelial eos/hpf (or 60 Eos/mm^2^). The symptom complex “esophageal dysfunction” includes specific symptoms, such as dysphagia or bolus obstruction but also unspecific symptoms, such as heartburn, regurgitation, food avoidance, chest pain or vomiting [25, 30]. A PPI trial is no longer necessary for the diagnosis of eosinophilic esophagitis [25].

The threshold for the diagnosis of eosinophilic esophagitis was empirically set at at least 15 eos/hpf [30]. In the presence of submucosal portions in the biopsy specimen, eosinophils in this area should be included in the count especially in cases of mucosal eosinophilic counts below the threshold [25]. If an appropriate number of biopsies are obtained, a sensitivity of 100% and specificity of 96% can be reached with this threshold. Normally, the esophagus is devoid of eosinophils, so any elevated number of eosinophils indicates a pathological process [31]. Other features such as basal zone hyperplasia, dilated intercellular spaces, eosinophilic micro-abscesses, eosinophilic degranulation, eosinophil surface layering, papillary elongation and lamina propria fibrosis are not specific for EoE but are pathological features that have been described in EoE [32, 33].

An eosinophilic eosinophilia (≥ 15/hpf) without any symptoms is defined as asymptomatic esophageal eosinophilia (aEE) [34]. To date it is not clear whether this phenomenon is transient and whether an aEE should be regarded as a precursor of EoE and is associated with a higher risk of developing EoE or complications such as fibrosis [34].

However, some patients may have adapted their eating habits and may be misclassified as aEE if not specifically asked about subtle symptoms of dysphagia. These patients should be asked about the liquid intake during meals, prolonged mealtimes, avoidance of hard textures, excessive chewing or inability to swallow pills [35, 36].

Although evidence is currently lacking the authors agree on recommending a clinical and histological follow-up after 6–12 months. If there are still no symptoms clinical and histological re-evaluation should be performed every 1–2 years. In cases of fibrosis or stenosis a clinical re-evaluation should be performed after 3 months and histological after 12 months [34]. This section will be updated in the future once more data are available.

Although other conditions may cause esophageal eosinophilia (gastroesophageal reflux disease, non-EoE EGID, achalasia, hypereosinophilic syndrome, connective tissue diseases, infections, autoimmune disorders or vasculitis, dermatological diseases with involvement of the esophagus, such as lichen planus ruber or mucous membrane pemphigoid, Crohn’s disease, pill-induced esophagitis, drug hypersensitivity reactions or graft versus host disease) and should be excluded before a diagnosis of EoE, many of these diseases may be associated with EoE itself, are very rare or present with other features and symptoms not typical for EoE [25, 37]. Systematic screening for these conditions is not required; however, based on the individual patient’s history and symptoms additional test ruling out the abovementioned conditions should be considered.

### Recommendation 1.8

In cases of clinical suspicion of EoE but not fulfilling the histological criteria of at least 15 eos/hpf, the biopsy samples should be re-evaluated by an expert in GI pathology. A sampling error should be suspected as well as a potentially underlying EoE variant.

In cases of high clinical suspicion with or without endoscopic features, an expert pathologist should review the specimen [38]. In around 20–40% of cases with an eosinophilic count between 1–14 eos/hpf, a second expert pathologist would reclassify the eosinophilic count as ≥ 15 eos/hpf [38, 39]. Especially other features such as basal zone hyperplasia, dilated intercellular spaces, eosinophilic micro-abscesses, eosinophilic degranulation, eosinophil surface layering, papillary elongation, and lamina propria fibrosis should be evaluated. Furthermore, a CD3 staining to accurately assess lymphocytic infiltration and diagnose/exclude lymphocytic esophagitis, should be performed [40].

In case of persisting clinical suspicion of EoE, the endoscopy with multiple biopsies should be repeated by an endoscopist experienced in EoE and the case should be discussed with pathologists and clinicians experienced in EoE. Despite the fact that the knowledge of EoE variants such as EoE-like disease, non-specific esophagitis and lymphocytic disease is growing [39], further studies will shed light on these variants.

### Recommendation 1.9

Endoscopic features of EoE are edema, rings, exudates, furrows and strictures. These signs should be evaluated with the EREFS score. The most affected area should be documented in the endoscopy report.

Eosinophilic esophagitis has typical endoscopic features like trachealization (rings), swelling and loss of vascular pattern (edema), patchy white plaques (exudates), longitudinal cracks (furrows) and strictures. Only 7–10% have no macroscopic changes [24]. A standardized score (EREFS score) was established by Hirano et al. with a good to moderate interobserver variability [41]. In the modified EREFS score, points are given to different features with a total score of 8: edema (0–1), rings (0–3), exudates (0–2), furrows (0–1) and strictures (0–1). The minor feature “crepe-paper esophagus” is in most studies not taken into account. The EREFS score can be used to assess severity of the disease and treatment response [42]. Furrow, edema and exudates represent the acute inflammatory process whereas rings and strictures symbolize fibrotic changes [43, 44]. An EREFS score ≤ 2 points is a good indicator for clinical and histological remission and is now defined as endoscopic remission [45, 46].

### Recommendation 1.10

Allergy testing with a skin prick test (SPT), atopy patch test (APT) or antigen specific IgE antibodies is not recommended to identify the culprit food allergen.

In adults, an allergy testing elimination diet shows an efficacy of histological remission of 26–35% [47]. Newer data support the lack of association of allergy tests and the culprit food in EoE [48]. Therefore, skin prick or patch tests and blood tests are not recommended to identify the responsible food allergen.

### Recommendation 1.11

Blood or urinary tests in eosinophilic esophagitis are not generally recommended due to low sensitivity or specificity for diagnosis of eosinophilic esophagitis.

The most assessed marker is peripheral eosinophilic counts in blood samples. Elevated levels could only be found in 34–50% of active EoE patients [49]. There are signs that normal (not activated) eosinophils differ in control patients and patients with EoE (40% vs. 32%). Also, a higher expression of cyclooxygenase‑2 can be found [50]. Elevated IgE levels show the atopic component in EoE patients; however, as EoE is not an IgE-mediated disease, there is no correlation of IgE levels with disease activity [51]. Other serum markers like interleukin (IL)-5, IL-33, eotaxin‑3, thymic stromal lymphopoietin (TSLP) and many others are not reliable in detecting active disease [52].

New markers like eosinophilic progenitor cells [53, 54], a new panel of blood mRNA levels containing CD274, CD101, CXCR6, TCRδ, Jα18, and FCεRII [55, 56] or urine 3‑BT level in creatinine [57] may be used to detect or monitor EoE patients in the future. To date, no non-invasive parameter is reliable enough to replace histological re-evaluation.

In cases of suspicion of an EGID, besides extensive biopsies as stated in recommendation 1.6, the abovementioned tests may be useful in the further evaluation.

### Recommendation 1.12

EoE and GERD are two distinct disease entities that can co-exist in one individual patient and can impact each other.

The EoE and GERD are different diseases that can coexist independently or influence each other bidirectionally. In the past, the definition of EoE has been changed several times and a strict separation between GERD and EoE was initially required; however, a clear distinction between EoE and GERD is not always possible or useful, as a patient can have both entities [58]. The close relationship between EoE and GERD is reflected by the following aspects: I) pathological pH-metry is found in up to 40% of EoE patients [59], II) up to 5% of all refractory reflux patients actually have EoE [60] and III) acid-inhibiting therapy with PPI can be effective for both GERD and EoE [58, 59].

There are 4 different possible scenarios that illustrate the overlap between EoE and GERD: 1) esophageal eosinophilia is caused by GERD but a lower number of eosinophil granulocytes are usually found compared to EoE [58, 61]. 2) GERD and EoE coexist but are not associated with each other. 3) gastroesophageal reflux contributes to the development of EoE: the pathomechanism of EoE is not yet fully understood. It is possible that a barrier dysfunction of the esophageal squamous epithelium leads to activation of the epithelial cells as they come into contact with allergens. GERD could cause or promote this barrier dysfunction and thus contribute to the development of EoE [58]. 4) EoE causes/exacerbates GERD: on the other hand, EoE can enhance the occurrence of GERD. Eosinophilic granulocytes release cytokines that affect the smooth muscles of the esophagus. Besides, in the course of the disease fibrosis of the esophagus can impair esophageal motility. Studies show that up to 20–70% of EoE patients have an impaired HR manometry, with the majority showing an ineffective motility disorder [62, 63]. Consequently, impaired clearance function in EoE can promote the development of GERD [58, 63].

## Therapy

### Recommendation 2.1

In all patients with the diagnosis of EoE an induction therapy should be started.

EoE is a chronic immune-mediated inflammatory disease with a progressive course leading to an esophageal remodeling and functional impairment [64, 65]. In order to prevent long-term complications such as fibrostenosis or food impaction and to improve quality of life, an appropriate treatment of the underlying inflammation is necessary [66, 67]. A prolonged diagnostic delay and untreated disease over time is the main risk factor for developing esophageal complications [44, 68]. Effective anti-inflammatory treatment showed the ability to improve clinical outcome and endoscopic findings [69]. Therefore, an induction therapy should be started in all patients with a diagnosis of EoE.

### Recommendation 2.2

**Fig. 1:**
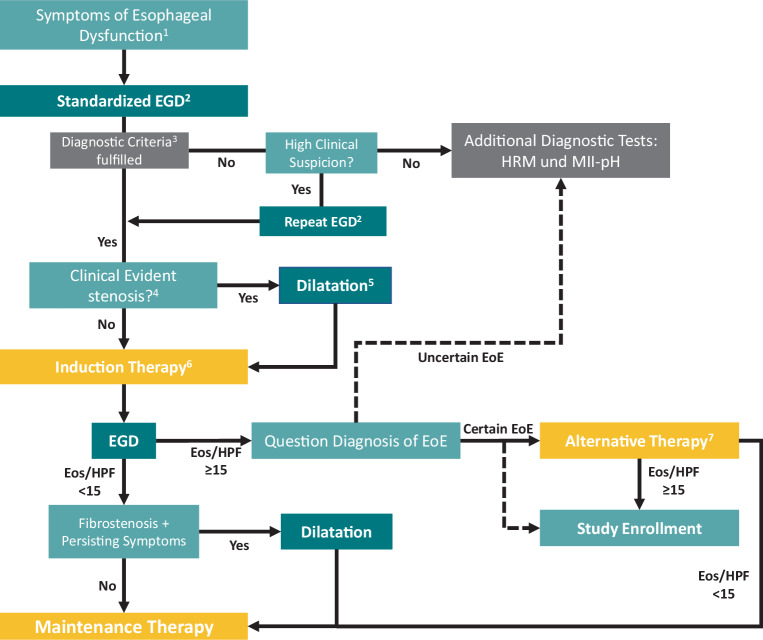
Therapy algorithm for induction therapy of eosinophilic esophagitis (EoE). ^1^bolus impaction, bolus sensation, dysphagia, odynophagia, noncardiac chest pain, heartburn (refractory to standard therapy), regurgitation, adaptive eating behavior, trouble swallowing pills; ^2^at least 6 biopsies from 2 different levels of the esophagus (ideally 4 quadrant biopsies of all 3 levels), evaluation of endoscopic activity with EREFS score, cessation of Proton Pump inhibitor (PPI) for at least 3 weeks, considering EoE variants; ^3^symptoms of esophageal dysfunction and positive histology (≥ 15 eosinophilic granulocytes/high power field (eos/hpf)); ^4^no passage with a standard gastroscope possible or bolus obstruction at first diagnosis with high risk of recurring impaction, consider low sensitivity of optical diagnosis of stenosis; ^5^target diameter ≥ 16 mm; ^6^firstline induction therapy: swallowed topical corticosteroids (STCs) 1 mg twice daily for 6–12 weeks; ^7^referral to EoE experienced institution; alternative therapies: first line therapy dupilumab 300 mg weekly for 24 weeks, second line therapy (Es)omeprazol 40 mg twice daily or elimination diet for 12 weeks. Abbreviations: *EGD* Esophagogastroduodenoscopy, *Eos* eosinophilic granulocytes, *HPF* high power field, *HRM* high resolution manometry, *MII-pH* impedance manometry

Therapeutic options for treating EoE are swallowed topical corticosteroids (STCs), high dose proton pump inhibitors, dupilumab or an elimination diet. A therapeutic algorithm as described in Fig. 1 can be used as guidance for induction therapy.

### Recommendation 2.2.1

Swallowed topical corticosteroids (STCs) are effective in achieving clinical histological remission and may be considered as first line induction therapy for 6–12 weeks. As maintenance therapy STCs can be safely used as long-term treatment.

After the first positive placebo-controlled trial of STC in the treatment of EoE in 2010 [70], many other small trials with different topical corticosteroids (budesonide, fluticasone) and different formulations (nebulized, suspension, orodispersible) confirmed the efficacy and safety of STC for induction of histological remission in eosinophilic esophagitis [71–74]. Two studies in children with systemic corticosteroids showed no significant difference in histological or symptomatic response but a higher incidence in adverse events [75, 76].

As scintigraphy studies showed poor contact time and lesser effect on eosinophilic count with nebulized corticosteroids in comparison to a viscous formulation, the mode of drug delivery is key in treating EoE [77]. After achieving up to 100% histological remission rate in a phase 2 trial with a budesonide orodispersible tablet formulation (BOT) [78], the EOS‑1 study [79] with a dosage of 1 mg budesonide twice daily was able to induce clinical histological remission in 58% vs. 0% in placebo after 6 weeks or in 85% vs. 0% after 12 weeks of an prolonged open-label treatment. A histological remission was achieved in 93% of patients [79]. A maintenance therapy with BOT with 1 mg twice daily or in the reduced dose of 0.5 mg twice daily was able to keep deep remission in 75% and 73.5% of patients for 48 weeks, respectively. There was no clinical relapse in 89.7% in comparison to 39.7% in the placebo group and no histological relapse in 86.8% of patients in comparison to 10.3% [4]. Until now, BOT is the only approved STC in the treatment for EoE.

In the FLUTE trial, a phase 2b study with a fluticasone propionate oral disintegrating tablet, a histological response was shown in 80% with 3 mg twice daily, 67% with 3 mg at bedtime, 86% with 1.5 mg twice daily and 48% for 1.5 mg to bedtime in comparison to 0% in placebo at week 12. At week 52 remission was maintained in 79% with 3 mg twice daily, 64% with 3 mg at bedtime, 89% with 1.5 mg twice daily and 30% for 1.5 mg to bedtime. A high percentage of candidiasis in patients with double administration may favor the intake of the tablet once daily to bedtime [80]. The phase 3 of the FLUTE trial is currently recruiting.

The most common side effect of STCs is oral or esophageal candidiasis with around 5–16%. In adults, no relevant signs of systemic corticosteroid effects were shown to date [4, 78, 79, 81].

In summary the treatment of EoE with STCs is not only highly effective in inducing remission in symptomatic patients but more able to maintain this effect in a high percentage with few side effects. We recommend using STCs as a first line induction therapy in most patients.

### Recommendation 2.2.2

In patients with simultaneous GERD or other indications for acid suppression PPI should be considered as induction and maintenance therapy.

PPI therapy is an unlicensed off-label therapy for the treatment in EoE [82]; however, PPIs are the most commonly prescribed first-line therapy for EoE due to their accessibility, low cost, and safety profile [82]. Although PPIs may be considered as effective in inducing remission in patients with EoE, it has to be kept in mind that most data are coming from observational studies and only two small randomized controlled trials exist with 25 and 42 patients, respectively [59, 83]. There are several systematic reviews and meta-analyses of randomized controlled trials which provided evidence that PPIs given at double dose led to histological remission (defined as < 15 eos/hpf) in 50% and symptomatic improvement in 60% of patients, irrespective of patient age, study design, or type of PPI evaluated [74, 82, 84]. Although the mechanism of action of PPI in EoE is not completely understood, the anti-inflammatory effects are not only dependent on gastric acid secretion inhibition. In biopsies from patients with EoE, downregulation of esophageal gene expression of eotaxin‑3 and TH2 cytokines, like interleukin (IL)-5 and IL‑3, was found for PPI, similar to that of patients treated with STC [85].

A recent systematic review and network meta-analysis compared the efficacy of esomeprazole, STCs, and biologics versus each other, or placebo, in terms of failure to achieve histological remission, symptomatic or endoscopic response in active EoE. In this analysis STCs ranked higher than PPI therapy for the induction of histological remission [86].

Currently there is no first-line therapy and treatment decisions should use a shared decision-making model based on efficacy, cost, ease of administration and patient preferences.

However, we recommend PPI treatment for induction therapy in patients with simultaneous GERD and/or other indication for PPIs. For all others, we tend to recommend STC due to higher efficacy. Additionally, in the presence of a stricturing phenotype, PPI therapy seems to be less effective to induce and maintain remission. Thus, treatment with STCs is preferred in this patient group even in presence of GERD. If necessary, a combination therapy for treatment of reflux symptoms and lesions (reflux esophagitis) should be considered.

The majority of published studies included 8 weeks of treatment with omeprazole 20–40 mg twice daily or equivalent to induce EoE remission. PPI treatment was most effective in achieving clinical and histological remission when used in double or higher, rather than standard or lower, doses (51% vs. 36%), and when the duration of therapy was prolonged from 8 to 12 weeks (50% vs. 65%) [84].

Consequently, we recommend a therapy of omeprazole 20–40 mg twice daily or equivalent for 12 weeks for induction of remission.

Once a PPI response has been achieved, there are limited data about long-term outcomes [87–90]. If remission induction with PPI is successful, dose reduction should be attempted. Among patient responders to PPI, a dose reduction was effective in maintaining EoE in histological remission (< 15 eos/hpf) in 69%, with 60% having < 5 eos/hpf. With respect to symptoms, 72% of patients under standard or lower doses of PPI were maintained in clinical remission and 13% of patients had lost response in 1–2 years [91]. Therefore, the long-term strategy is to use the minimum effective dose to maintain remission, usually standard PPI doses. After proof of clinical and histological efficacy, the minimum dosage should be titrated.

### Recommendation 2.2.3

Elimination diet can be considered as induction and maintenance treatment. If this treatment option is considered, a consultation of a dietician is mandatory. Due to less restriction and improvement of quality of life a 1FED (animal milk) or a step-up approach with 2FED (animal milk and wheat/gluten) is preferred.

As EoE is an immune-mediated disease triggered by a food antigen and rarely by an inhaled allergen [92–94], the only therapeutic option to treat the cause of EoE is to eliminate the causative allergen.

There are theoretically three approaches to eliminate food allergens in EoE: elemental diet, allergy testing elimination diet and empirical elimination diets.

The elemental diet in which only allergen-free liquid formulas can be consumed by the patient, is the most effective dietary option. Clinical remission can be induced in up to 90% of all patients [47, 71, 95–97]; however, data in adult patients are sparse. There is just one small study with 18 patients which showed an efficacy of 94% of histological improvement [95]. Despite the high efficacy, this treatment option as long-term treatment is not tolerable due to the expensive costs, the undesirable taste and the massive impact on patient daily life. This option should only be considered in highly symptomatic patients without any therapeutic alternatives.

As described in statement 1.10 allergy testing elimination diet is not recommended in patients with EoE.

The empirical elimination diet is mostly used as dietary treatment option of eosinophilic esophagitis. In the 6 food elimination diet (6FED) the most common food allergens milk, wheat/gluten, egg, soy/legumes, peanuts/tree nuts and fish/shellfish are avoided. In older studies up to 70% of patients achieved histological remission and a symptom reduction in up to 94% patients. After cessation of dietary restrictions the mucosal eosinophilic count returned to the prior status [98]. Many following studies concluded that the most common trigger of EoE is cow’s milk (60–74%) followed by wheat (28.6–50%), soy/legumes (10–23.8%), peanut/tree nuts (6–21.9%), egg (5–27.6%) fish/shellfish (0–19%) [98–101]. There are heterogeneous results if there is a single food trigger (35.71–72%) or two (up to 30.95%) to three (33.3%) triggers [99, 100]. Overall, the elimination diets were heterogeneous in the definition of the food components, so some studies excluded only cow’s milk while others excluded every dairy product (including goat and sheep milk due to cross-reactivity). This was the same with wheat and gluten-containing grains or soy and legumes. Therefore, a very strict approach with elimination of every dairy product and all gluten-containing grains is recommended with the possibility of reintroducing certain components after assuring histological remission.

In systemic reviews this treatment option seems to have an efficacy of 67.9–72.1% [47, 71]. To avoid long-term restrictions of food components which may not play a role in the pathogenesis of EoE in the individual patient, a systematic reintroduction is performed with endoscopic follow-up every 6 weeks. This can lead to a time span of a median of 16.8 months and 5.6 endoscopies [99]. This approach is very time consuming, expensive and many endoscopic procedures are necessary for one patient.

To reduce the burden of dietary restrictions and the need of multiple endoscopic evaluations, a step-up approach beginning with an elimination of milk and gluten (2FED), followed by a 4FED and 6FED in nonresponders was introduced [71, 101, 102].

However, the largest prospective randomized dietary study in EoE raised new questions and indicated that the remission rates in earlier studies with 6FED have been overestimated. In this multicenter randomized, open-label trial with 129 patients, no statistical difference of inducing histological remission could be demonstrated with a 1FED with milk (34%) compared to a 6FED (40%; *p* = 0.58). Due to minor restrictions in milk exclusion diet compared to 6FED this seems a favorable therapy option, although the effect of 6FED seems to be disproportionally low in comparison to other studies [48].

In conclusion, we only recommend dietary treatment in patients with a high motivation due to major implications for daily life (strict dietary restrictions especially eating outside in restaurants, at work or at social gatherings) or in patients with a histological non-response to medications. Dietary treatment should be accompanied by a specially trained dietician who is familiar with the treatment of EoE [103, 104].

Due to lesser restrictions, but the same efficacy, we recommend starting with a 1FED for 12 weeks with endoscopic assessment of histological response. The widening of the endoscopic follow-up from 6 to 12 weeks in contrary to the aforementioned studies was decided because of uniformity of control intervals. In the case of a non-response, a 2FED or even a 4FED can be tried. If remission is achieved with a 2FED or 4 FED reintroduction of food components can be done.

### Recommendation 2.2.4

Dupilumab can be considered as induction and maintenance treatment in EoE. Mostly used in patients refractory or intolerant to STC, it may be used as first line therapy in patients with other clinically relevant type 2 inflammatory diseases or contraindications for corticosteroids.

The inflammatory process in EoE is caused by an antigen-triggered type 2 inflammation in which IL 4 and 13 play a role in propagating the inflammatory cascade. Activation of IL-13 causes barrier dysfunction and infiltration of immune cells like eosinophils, mast cells and TH2 helper cells. IL‑4 is additionally involved in differentiation of TH2 helper cells and chemotaxis of eosinophilic granulocytes [105]. Dupilumab is a monoclonal human antibody which blocks the receptors for IL 4 resulting in a IL‑4 and IL-13 blockage. Dupilumab is already used in allergic asthma, atopic dermatitis and chronic rhinosinusitis with nasal polyps.

After a phase II trial in 47 adult patients refractory to PPI demonstrated improvement in histological, endoscopic and symptomatic endpoints [106], the phase III trial could confirm the efficacy and safety of dupilumab in EoE. In the part A group, a histological remission (< 6 eos/hpf) was achieved in 60% of the dupilumab weekly treatment vs. 5% in the placebo group as well as a significant reduction in dysphagia (dysphagia symptom questionnaire, DSQ) after 24 weeks of treatment [5]. Although similar findings could be observed in the part B study in which an additional dose (dupilumab every second week) was administered, only patients with the weekly dupilumab administration showed a significant reduction of symptoms compared to placebo [5].

The extension phase (part C, 52 weeks) of part B with more patients was published in a second paper (Liberty EoE Treet Study). The primary endpoint (< 6 eos/hpf) was reached in 85% of patients treated with dupilumab weekly from the beginning and in 100% if a cut-off of < 15 eos/hpf is considered. Of patients who had not reached histological remission after 24 weeks, 64% achieved this goal after 52 weeks in the weekly dupilumab group. In the extension phase no placebo group was included. The reduction in symptom scores was higher in the weekly dupilumab groups in comparison to the administration every second week [107]. These study results seems to be transferable into real life [108].

The treatment with dupilumab can be considered safe due to low level of adverse events in approval studies over multiple indications. The main adverse events were reactions at the injection site (10–12%) and a slightly increased risk of upper respiratory tract infections [5]. The higher incidence of conjunctivitis was only observed in atopic dermatitis patients [109].

As the study population of the phase III trial was refractory to STC in around 60–80%, 30–40% had a clinical history of dilation and about 40% had already tried elimination diets, no conclusions can be drawn regarding the efficacy against STC. Hence, the position of dupilumab in the algorithm of the treatment of EoE has to be determined [110, 111]. Therapy costs have to be taken into account as well as other type 2 inflammatory diseases that may be treated simultaneously with one medication.

However, we recommend using dupilumab in patients refractory or intolerant to STC or with other concomitant type 2 inflammatory diseases that may need a biologic therapy.

### Recommendation 2.3

After 12 weeks of induction therapy, an endoscopic follow-up with biopsies is necessary to demonstrate histologic response.

The inflammatory activity only correlates moderately with clinical symptoms or endoscopic findings. Neither clinical symptoms nor endoscopic findings are adequate tools to assess response or remission after induction therapy [42, 112, 113]. A systematic review and meta-analysis showed a moderate reliability between improvement of clinical symptoms and histological remission [114].

Reliable noninvasive or minimally invasive diagnostic tools to monitor the disease activity are lacking at the moment; however, some minimally invasive tests (e.g., Cytosponge^TM^ (Cambridge University Hospitals NHS Foundation Trust [legal manufacturer]; produced by Europlaz, Essex, UK), string test) showed promising results in early studies. As these tests are not validated in larger studies and approved for clinical use, more investigations are necessary [115, 116].

Therefore, an endoscopic re-evaluation with biopsies for histological assessment is recommended in all patients 6–12 weeks after the start of induction therapy.

### Recommendation 2.4

In order to avoid long-term complications such as fibrosis, to improve quality of life and to reduce the risk of esophageal food impaction, maintenance therapy is mandatory.

The natural course of the EoE is a progressive course from an inflammatory to a mixed inflammatory/fibrostenotic phenotype in most patients. Eosinophilic infiltration of esophageal tissue persists over time and leads to subepithelial fibrosis in the absence of an adequate treatment [44, 117]. The duration of an untreated EoE is the driving risk factor for esophageal fibrosis and stricture.

Relapse occurs in nearly all patients withdrawing from therapy [118, 119]. A maintenance therapy is able to avoid long-term complications such as fibrosis [120], to improve quality of life [121] and to reduce the risk of esophageal food impaction [43]. For these reasons, we cannot recommend withdrawal from therapy.

**Fig. 2:**
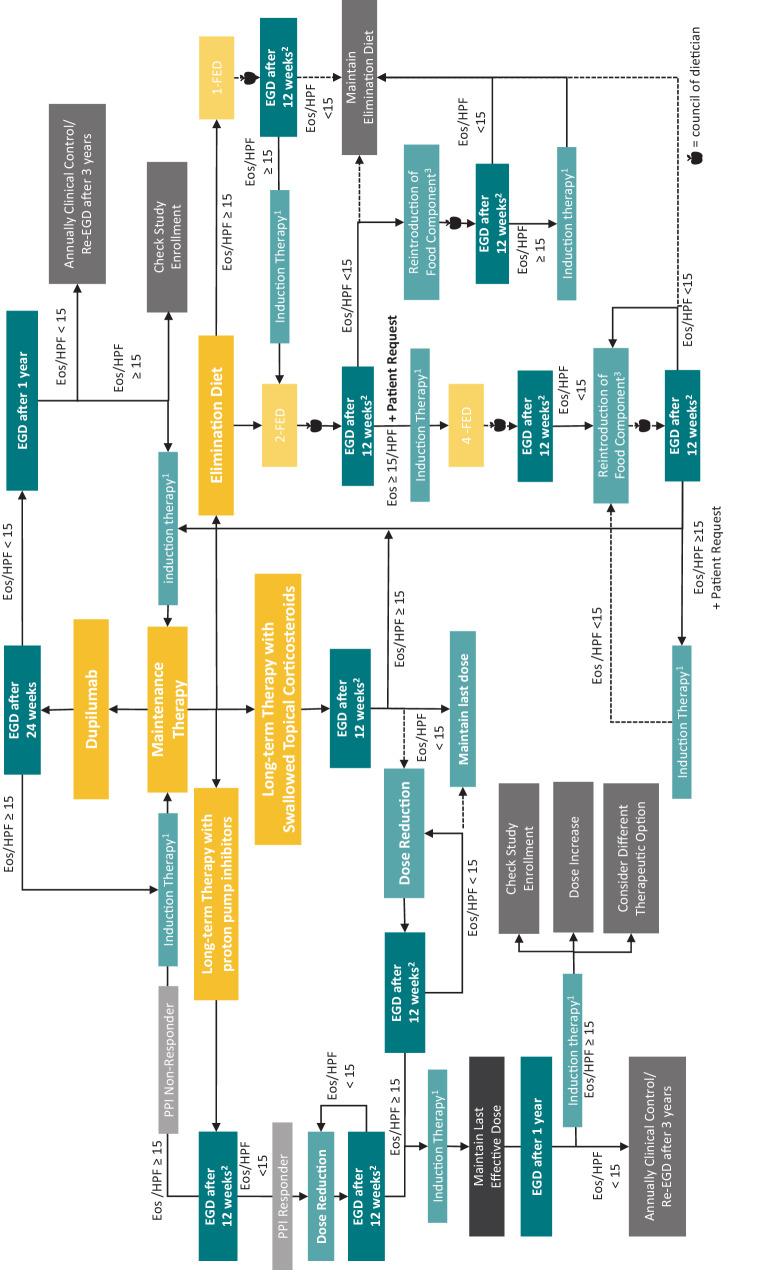
Therapy algorithm for maintenance therapy of eosinophilic esophagitis: ^1^First line induction therapy: orodispersible budesonide 1 mg twice daily for 6–12 weeks or last effective therapy; In cases of relapse after change in therapeutic management a shorter course of induction therapy for 6 weeks is possible; ^2^a prolongation of the interval to 24 weeks is possible in cases of patient request; ^3^consider multiple food reactions in food reintroduction scheme, if reintroduction of one food component does not maintain remission Abbreviations: *EGD* Esophagogastroduodenoscopy, *Eos* eosinophilic granulocytes, *FED* Food Elimination diet, *HPF* high power field, *HRM* high resolution manometry, *MII-pH* impedance manometry

All available therapies for induction therapy, PPIs, STC, dupilumab and diet regimens, can be used for maintenance of remission [5, 120, 122, 123]. A systematic therapeutic algorithm as described in Fig. 2 could guide decision making in maintenance therapy.

### Recommendation 2.5

In the majority of patients, combination therapies cannot be recommended. This option can be considered in selected cases with partial response to the first therapy.

An alternative to monotherapy may be a combined treatment strategy, the use of nutritional therapies in conjunction with other treatment modalities such as PPIs, topical corticosteroids, or biologic agents. In general, a combination therapy targets different aspects of the underlying pathophysiology of EoE [124]; however, there are very little data on the effectiveness of combination therapy and the available literature shows contradictory results. There are studies showing clinical improvement when dietary treatment is combined with STC and when a PPI is combined with STC, but without achieving histological improvement [125, 126]. In contrast, a recent retrospective study showed that a combination therapy of PPI and diet can bring EoE patients into histological remission who previously did not respond to monotherapy [127]. Furthermore, in most approval studies [5, 79] continuing the treatment with PPI was allowed, resulting in a combination therapy in some patients; however, the rationale behind this management is debatable because these patients clearly have not gone into remission with PPI.

Consequently, combination therapies cannot be generally recommended; however, in patients with EoE partially responding to the first treatment, a combined therapy can be an alternative to a switch of medication.

### Recommendation 2.6

After every major change of treatment, a clinical and histological re-evaluation should be performed to ensure maintenance of remission.

The inflammatory activity does not sufficiently correlate with clinical symptoms or endoscopic findings. Neither clinical symptoms nor endoscopic findings are adequate tools to assess response or remission [42, 112, 113]. A systematic review and meta-analysis showed a moderate reliability between improvement of clinical symptoms and histological remission [114]. Reliable noninvasive or minimally invasive diagnostic tools to monitor the disease activity are lacking at the moment. An endoscopic and histological evaluation should be performed when the patient reports a worsening of clinical symptoms or after a change in therapy to ensure maintenance of remission [128].

### Recommendation 2.7

Due to high risk of recurrence and esophageal food impaction, a trial of withdrawal of treatment is not recommended.

Eosinophilic infiltration of esophageal tissue persists over time and leads to subepithelial fibrosis in the absence of an adequate treatment [44, 117]. The duration of an untreated EoE is the driving risk factor for esophageal fibrosis and the risk of stricture increases by 9% for every year of untreated disease [129].

A symptomatic and histological relapse often occurs in patients withdrawing from therapy for EoE. In a retrospective analysis of adult patients at the Mayo Clinic, 61% experienced recurring symptoms that led to the need for repeated therapy [118]. Even in patients with deep remission (clinical, endoscopic and histological remission), clinical relapse occurred in 82% after a median of 22 weeks [119]. Data from clinical trials [4, 130] support this aforementioned high and rapid recurrence rate. Therefore, we cannot recommend withdrawal from therapy.

### Recommendation 2.8

Patients with STC-refractory disease should be evaluated and managed in institutions experienced with management of EoE.

To date, there has been no standard definition for refractory EoE. In most publications refractory EoE is defined as persistent symptoms, persistent esophageal inflammation on histology or endoscopy, or a combination of both after treatment for EoE [124]. This definition is not entirely consistent with diagnostic criteria that do not require endoscopic findings [131].

In cases of refractory disease, clinicians should first search for an alternative cause of esophageal eosinophilia, check patient compliance, appropriate route of medication and adequate dosage prior to changing therapy [124].

When a patient has persistent symptoms, endoscopic findings as well as esophageal eosinophilia, there is most likely a true nonresponse [131]; however, when there is discordance between symptoms, endoscopy and histology, additional investigation is necessary. This conflicting response may occur because of the different inflammatory and fibrostenotic aspects of EoE [131]. In cases of persistence of symptoms despite histological remission, we recommend a barium esophagram and/or an EndoFLIP^TM^ (Medtronic, Minneapolis, MN, USA) measurement to detect esophageal strictures not visible on endoscopy. In patients treated with STC candida esophagitis can cause odynophagia, which should be treated with antifungal agents. In addition, esophageal dysmotility as well as esophageal hypervigilance should be ruled out [124].

Data describing the success rates of second-line therapies are limited. Studies report response rates between 48% and 54% of initial nonresponders, who received second-line agents [132, 133]. In a European observational study 79% of patients who transitioned to STC achieved histological remission (eos < 15/hpf) compared with 65% of patients who switched to PPIs, and 39% of patients who switched to diet [134]. The PPI therapy seems to be less effective in inducing clinical and histological remission for patients who had previously failed diet or STC treatment than when used as a primary therapy [131]. The phase 3 trial of the human monoclonal antibody dupilumab, which blocks IL‑4 and IL-13, included patients who had not achieved remission after 8 weeks of PPI therapy. Here, weekly administration of dupilumab showed histological remission in 60% after 24 weeks [5]. Consequently, the change of therapy to dupilumab represented another therapeutic option.

However, patients who do not respond to any first-line treatment should participate in a clinical trial. [124] If none of the above options is possible, a therapy attempt with an elemental diet or a combination therapy may be considered [124]. Due to complex diagnostic evaluation and therapeutic regimens, we recommend referring these patients to institutions experienced in the management of EoE for a second opinion.

## Dilation

### Recommendation 3.1

In patients with clinically relevant fibrostenosis and with the necessity of dilation, a combination with anti-inflammatory treatment is required.

As esophageal dilation treats only fibrosis and not the underlying cause of the disease, patients after dilation without an anti-inflammatory treatment have a higher likelihood of recurrent dysphagia than patients having a maintenance therapy [135, 136]. A maintenance therapy with either STC, PPI, dupilumab or diet is therefore necessary.

### Recommendation 3.2

Regardless of the used technique, esophageal dilation is safe and effective in patients with EoE.

Many retrospective studies demonstrated the high efficacy of dilation in patients with EoE with perforation rates (0.38%) similar to non-EoE patients [137]. Through the scope balloon technique, bougie method [138] or an attachment cap (BougieCap (Ovesco Endoscopy AG, Tubingen, Germany)) [139] can be used as a treatment of patients with fibrostenotic EoE. Fluoroscopy is rarely needed and dilation is safe even in the case of ongoing inflammation. The target diameter is typically at least 16 mm, but dependent on the initial caliber of the lumen [140]. A mucosal disruption is an indicator of clinical response and is, in most cases, not associated with adverse events. As in other indications, the strategy “start low and go slow” should be endorsed with gradual dilation in multiple sessions in patients with a narrow-caliber esophagus or with a narrow stricture, even if evidence is lacking.

### Recommendation 3.3

In patients with persistent dysphagia despite histological remission and lack of a visible stricture an empirical dilation may be performed.

As endoscopy has a low sensitivity in identifying a narrow-caliber esophagus (cut-off diameter < 15 mm has a sensitivity of only 25%) [141], patients with dysphagia in histological remission may benefit clinically of an empirical esophageal dilation.

### Recommendation 3.4

If dilation is necessary, a luminal diameter of ≥ 16 mm should be aimed for to reduce long-term complications.

Despite the lack of prospective studies investigating the minimum esophageal diameter to improve symptoms and reduce complications in patients with EoE, most data demonstrate a better outcome in patients with an esophageal diameter of at least 16 mm. A diameter of at least 16 mm is associated with fewer endoscopic dilation sessions over 1 year in benign strictures [142]. Furthermore, in patients with EoE an improvement of esophageal diameter up to 16 mm is strongly associated with a decrease of dysphagia symptoms [139, 140] and the other way around, a diameter of < 17 mm is associated with a higher risk of esophageal food impaction [143].

## Esophageal food impaction

### Recommendation 4.1

Patients with EFI should not induce vomiting and should preferably attend an endoscopy unit/emergency department within 2 h.

Although data regarding the waiting time for spontaneous bolus resolution are lacking, most experts agree that patients should seek medical help within 2 h [9]. A longer EFI may be associated with a higher risk of esophageal rupture [144]. As vomiting can lead to increased intraluminal pressure and subsequently to esophageal rupture [144], vomiting should not be attempted. Older studies suggest that sparkling drinks may be tried to resolve EFI [145]; however, in patients with complete obstruction, the risk of aspiration may be increased.

### Recommendation 4.2

Patients with EFI should preferably have esophagogastroduodenoscopy within 6 h. Before EGD, radiological evaluation or medications to resolve EFI is not advisable.

Although some data show that complications, especially the risk of esophageal rupture, may increase with the duration of EFI [146, 147], newer data [148] do not support these earlier findings. Radiographic evaluation is unnecessary in uncomplicated nonbony EFI and may only delay urgent endoscopy. If perforation is suspected, a CT scan is the recommended method. Pharmaceutical management is mostly not useful and therefore not generally recommended [149]. Despite the common use of glucagon in many emergency departments, there is no evidence of the effectiveness in EFI resolution or shortening the endoscopy time when administering glucagon in patients with EFI [150].

### Recommendation 4.3

There is lacking evidence whether general anesthesia with endotracheal intubation or conscious sedation should be used in cases of EFI.

There is scarce data regarding the type of sedation in cases of EFI. Although endotracheal intubation may secure airways, one retrospective study demonstrated a higher rate of adverse events (AE) in patients having had elective intubation [151]; however, it can be hypothesized that patients who had intubation, had more difficult EFI and AEs were not related to intubation.

### Recommendation 4.4

Push or pull technique with or without endoscopic devices may be used depending on physicians’ expertise.

Studies demonstrate similar safety and efficacy of the push and pull technique in adults [152, 153]. Different endoscopic devices such as Roth Net® (STERIS, 5976 Heisley Road, Mentor, OH 44060, USA) retrievers, biopsy forceps, tripod forceps, polypectomy snare and alligator forceps may be used. A recently published prospective study demonstrated a higher rate of en bloc removal, a shorter procedural time and lower rate of adverse events with a cap-assisted pull-method compared to conventional techniques [154]. Although this technique may have benefits, it has to be mentioned that all patients in this trial had endotracheal intubation with secured airways.

### Recommendation 4.5

In patients presenting with EFI, sufficient biopsies of the esophagus should be taken during the index endoscopy to diagnose or exclude eosinophilic esophagitis.

As EoE represents the most common cause of EFI nowadays [6], it is of upmost importance to diagnose or exclude EoE at the index endoscopy; however, only the minority of patients presenting with EFI will have biopsies taken at the index endoscopy [155]. Patients not having had biopsies are more likely to be lost to follow-up resulting in persistence of symptoms and increased risk of having repeated EFI [156–158]. Furthermore, patients without relevant endoscopic findings at the index endoscopy are 7‑fold more likely to have an inappropriate follow-up [159].

As discussed in the previous statement (recommendation 1.4) a sufficient number of biopsies (three biopsies of the distal and three biopsies of the middle/proximal esophagus) is necessary to get a high diagnostic accuracy. Due to mechanical irritation of the mucosa resulting in unspecific inflammatory changes in the area of bolus impaction, these regions should be spared. In cases of stenosis or mucosal lesions suspicious for neoplasms, biopsies should be taken to rule out malignancy. In patients with diarrhea, abdominal pain or bloating, as stated in the previous section, extensive biopsies of the stomach and duodenum are needed. This might be difficult in the setting of emergency endoscopy. In this particular situation we recommend performing an additional endoscopy within 2–3 weeks without starting or changing current treatment as stated in recommendation 4.6.

### Recommendation 4.6

In patients with spontaneous resolution of EFI or in patients where sufficient esophageal biopsies have not been performed during index endoscopy, an endoscopy without PPI or STC should be repeated within 2–3 weeks.

Nearly all patients with a spontaneous resolution of the EFI will have an underlying esophageal disease and half of them will not receive adequate follow-up [160]. Therefore, it is necessary to schedule an endoscopy appointment at the outpatient clinic at the time of presentation. As PPI can be used to treat an underlying EoE and thus masking the diagnosis [26], PPI should not be started until sufficient biopsies have been taken. If PPIs have been started without adequate biopsies, they should be stopped at least 3 weeks before endoscopy; however, as no data exist regarding the duration of treatment effect after stopping PPI therapy, an EoE should not be ruled out definitely in patients with symptoms of EoE.

### Recommendation 4.7

Any patient presenting with EFI to the emergency department should receive an appointment within the next 4 weeks in the gastroenterology outpatient clinic.

In around 30% of EoE patients EFI represents the first contact with the healthcare system, so that these patients should not be lost. As up to half of the patients will not have a follow-up after EFI [157], it is necessary to implement standardized protocols. It is advisable to schedule the next appointment at the outpatient clinic at the time of EFI, because this approach will reduce loss to follow-up [156]. If it is not possible to make an appointment during the visit in the emergency department, the endoscopist performing the bolus removal or the physician/gastroenterologist involved in the case is responsible that the patient receives an appointment in an outpatient clinic within the next 4 weeks.

## Follow-up

### Recommendation 5.1

Patients with EoE in clinicopathologic remission should have clinical and endoscopic follow-up every 1–2 years.

As EoE is a chronic and progressive disease, a follow-up concept is necessary and important. Data suggest follow-up intervals of 12–18 months [161] that may be extended to every 24 months to detect relapsing disease early and to prevent fibrostenosis [162]. Symptoms correlate at best modestly to biologic disease activity [113], especially in patients having had dilatation of the esophagus [163]. Therefore, not only clinical but also endoscopic follow-up with biopsies is necessary to monitor EoE. A new tool to determine EoE activity at follow-up is the newly developed index of severity of EoE (I-SEE) [164]. This tool with its three domains (symptoms, inflammatory and fibrostenotic features) is able to classify disease severity as mild, moderate or severe. Using the I‑SEE in follow-up appointments, disease severity can be monitored and therapy can be adapted in the case of progression.

## Abbreviations

AE: Adverse event
aEE: Asymptomatic esophageal eosinophilia
ATP: Atopy patch test
CT: Computed tomography
EFI: Esophageal food impaction
EGD: Esophagogastroduodenoscopy
EoC: Eosinophilic colitis
EoE: Eosinophilic esophagitis
EoG: Eosinophilic gastritis
EoN: Eosinophilic enteritis
EMA: European Medicines Agency
Eos/hpf: Eosinophils per high power field
FA: Food allergy
GERD: Gastroesophageal reflux disease
I‑SEE: Index of severity of EoE
Non-EoE EGID: Non-eosinophilic esophagitis eosinophilic gastrointestinal diseases
OIT: Oral immunotherapy
PPI: Proton pump inhibitor
PPI-REE: Proton pump inhibitor-responsive eosinophilic esophagitis
SPT: Skin prick test
STC: Swallowed topical corticosteroids
TH2 Inflammation: Inflammation with Type 2 T-cells
TSLP: Thymic stromal lymphopoietin
